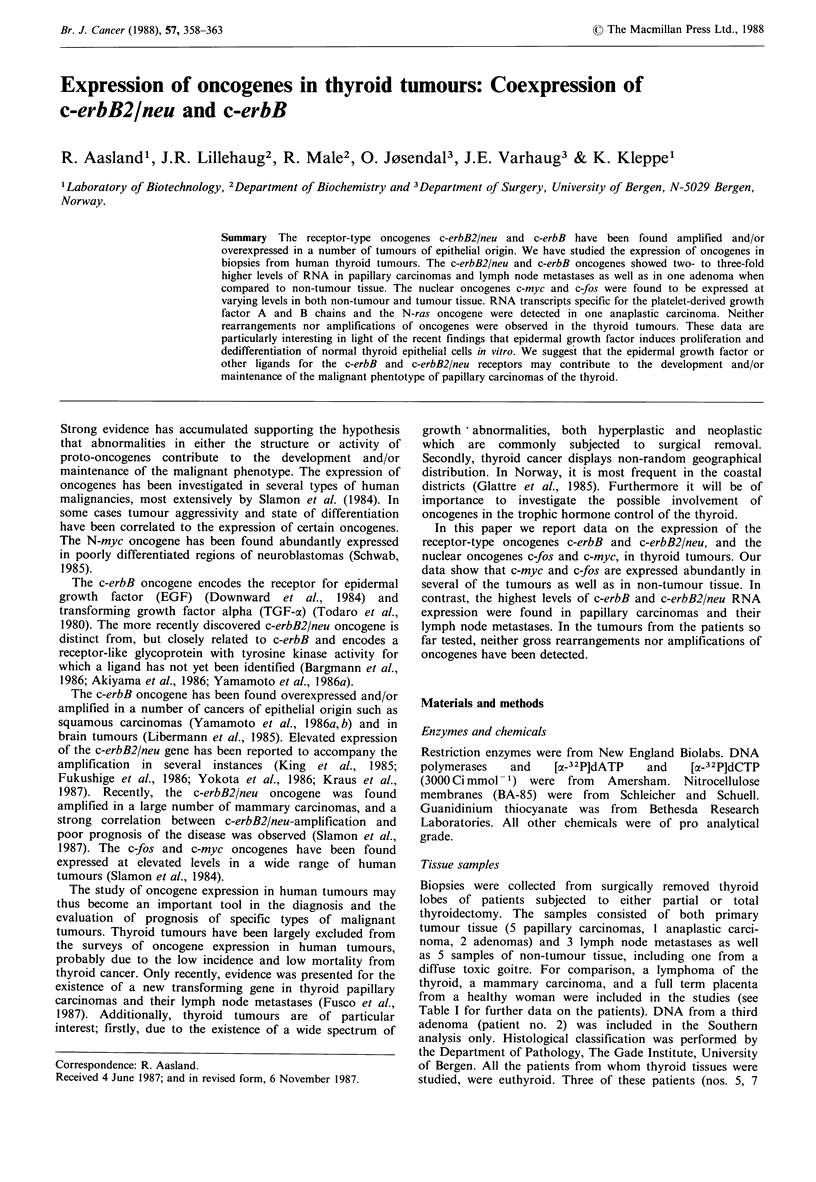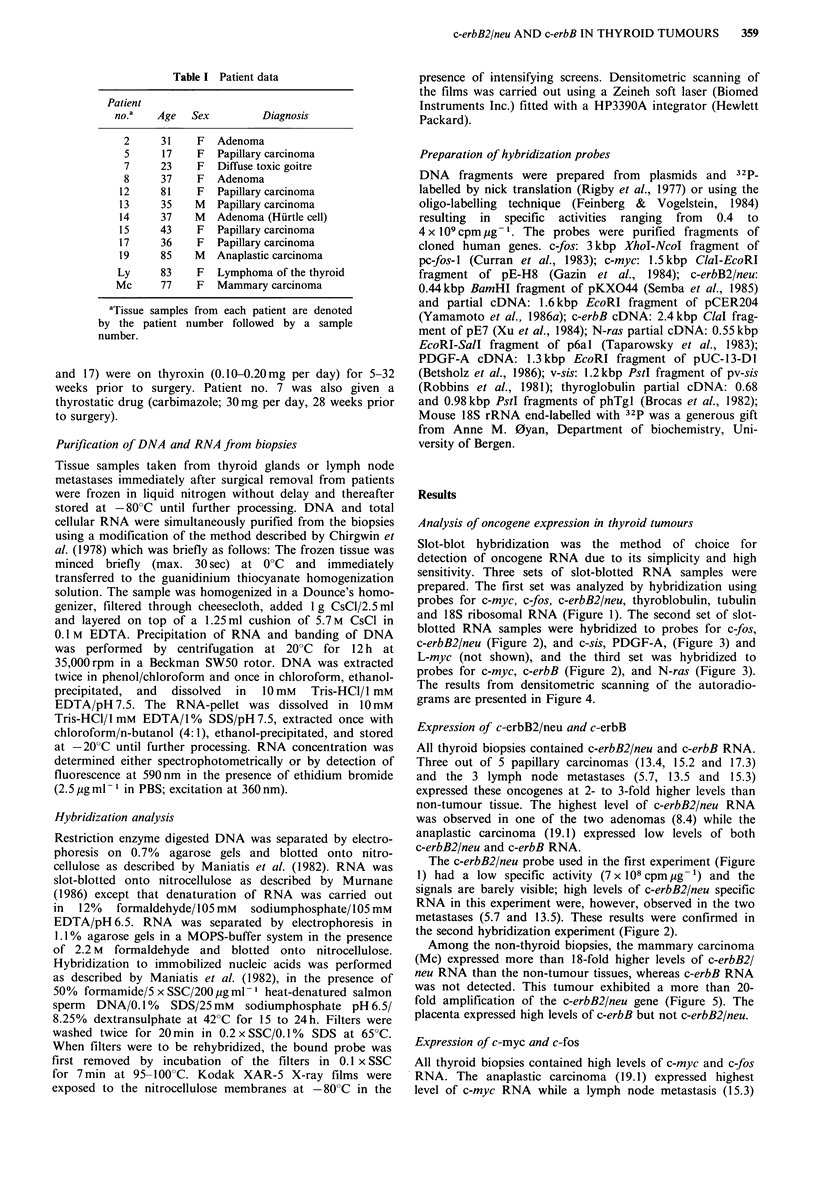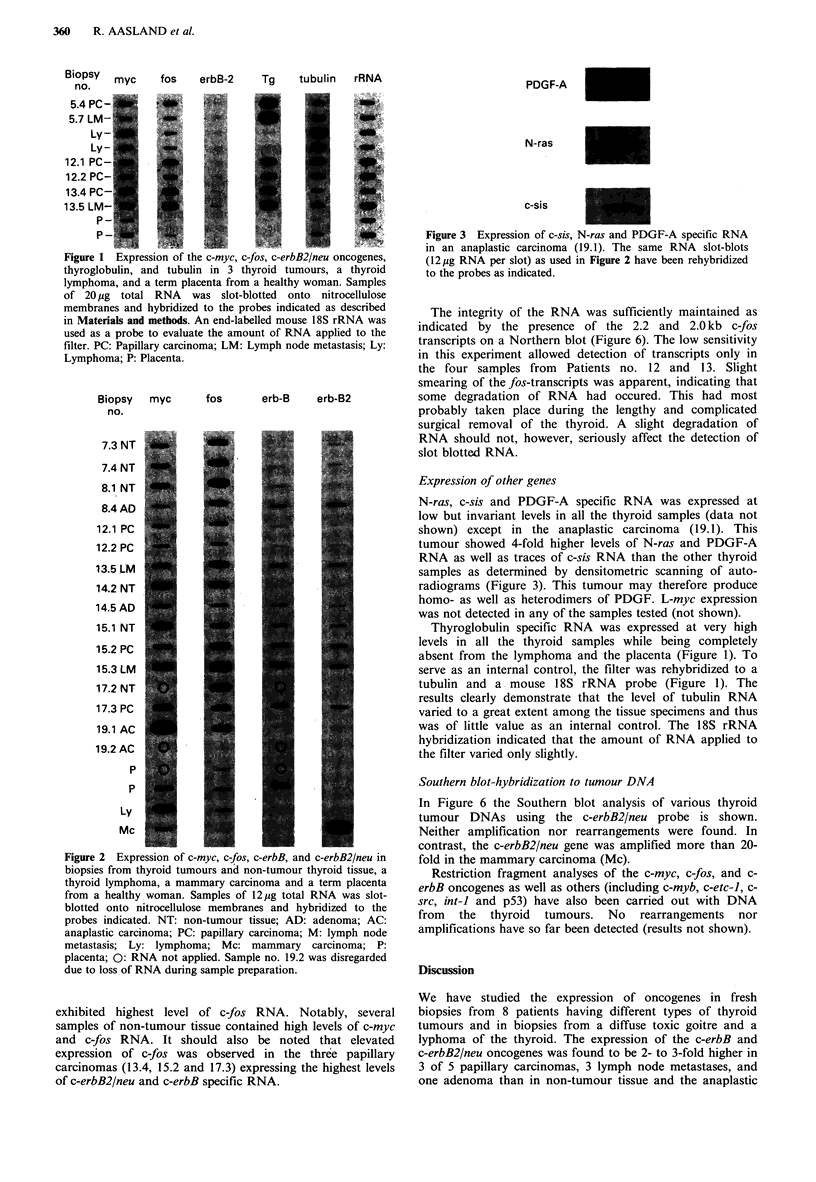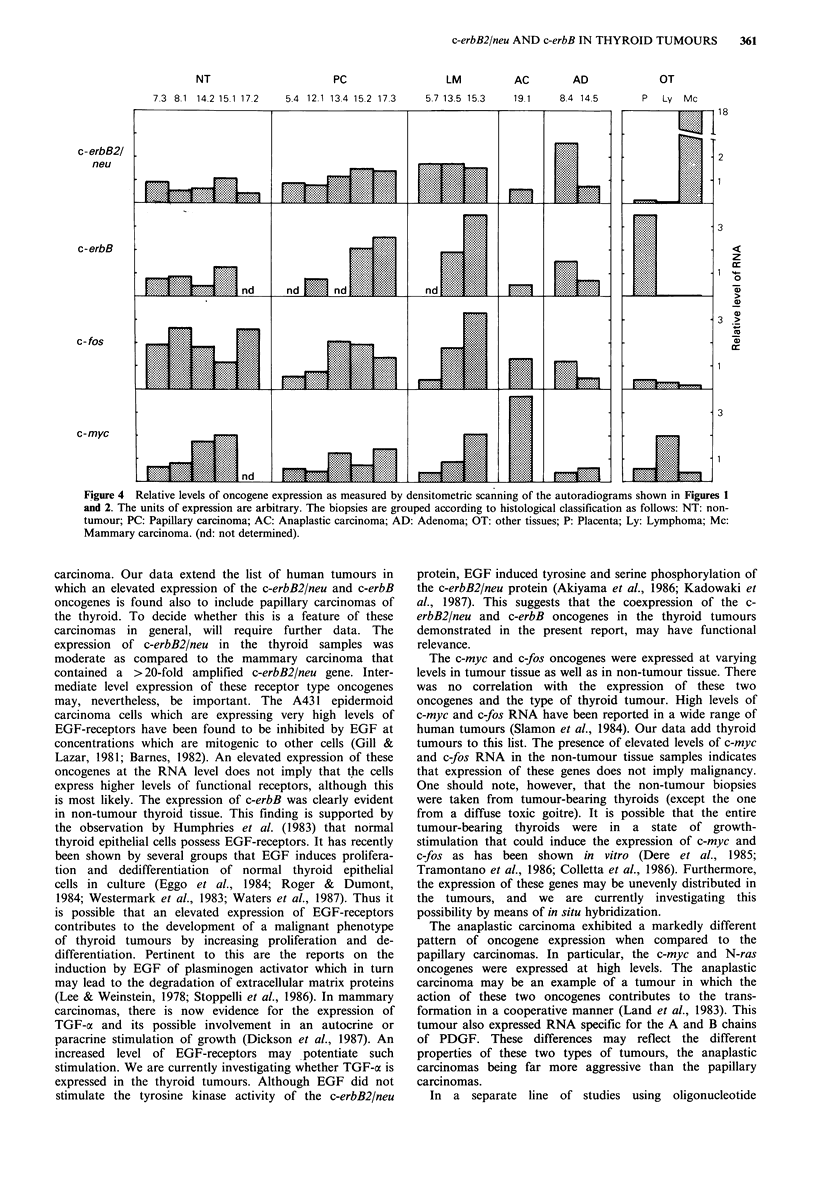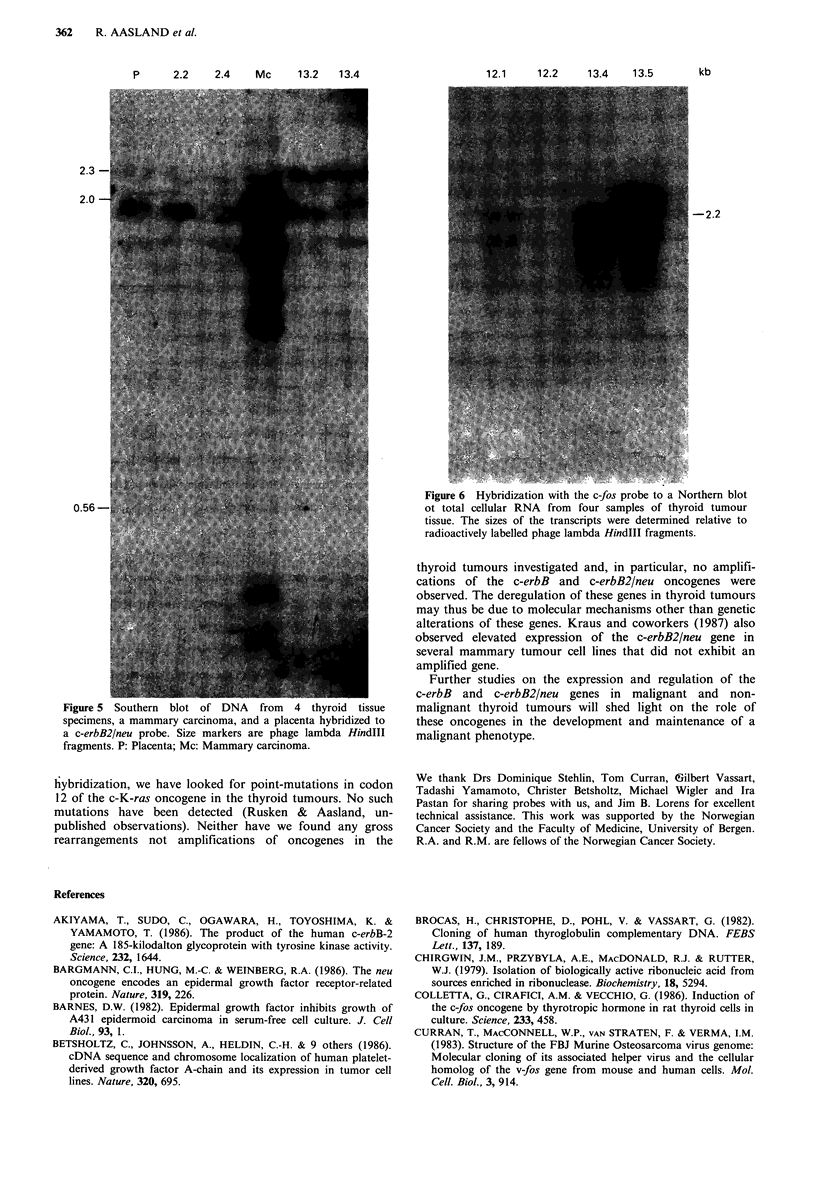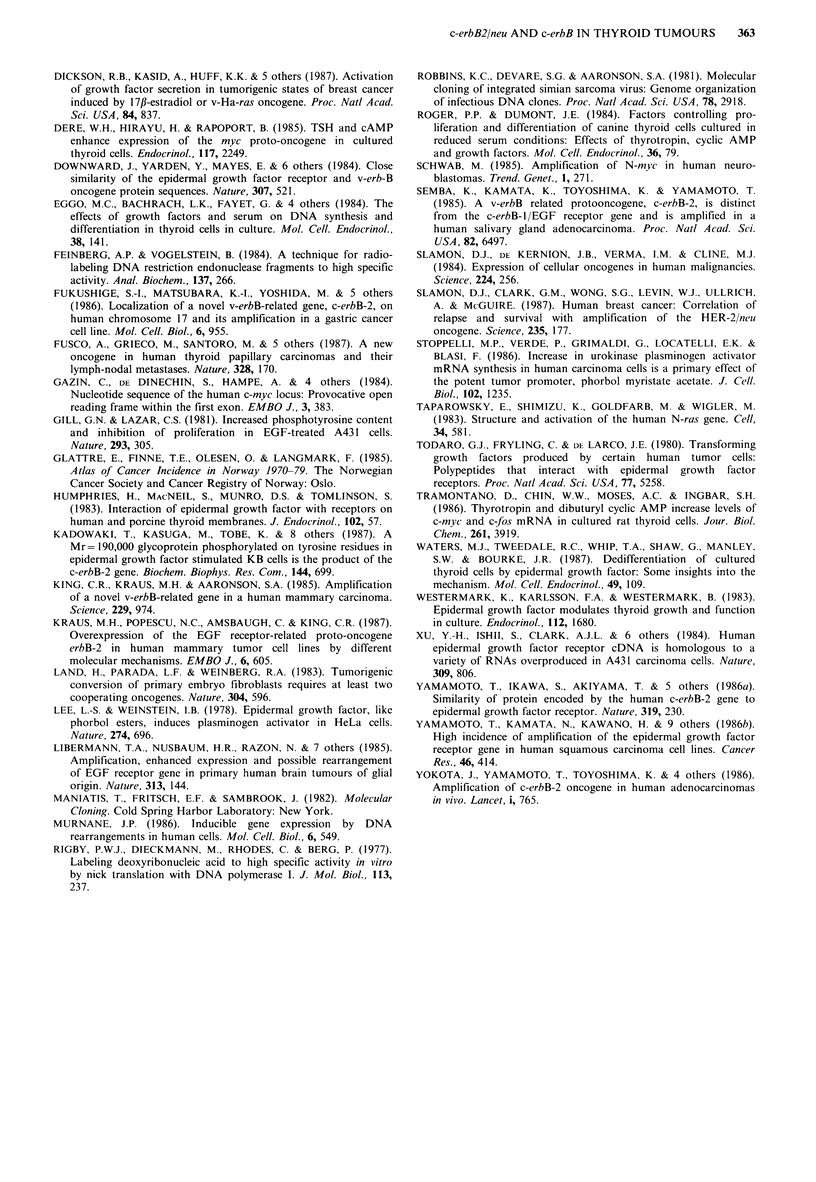# Expression of oncogenes in thyroid tumours: coexpression of c-erbB2/neu and c-erbB.

**DOI:** 10.1038/bjc.1988.82

**Published:** 1988-04

**Authors:** R. Aasland, J. R. Lillehaug, R. Male, O. JÃ¸sendal, J. E. Varhaug, K. Kleppe

**Affiliations:** Laboratory of Biotechnology, University of Bergen, Norway.

## Abstract

**Images:**


Br. J. Cancer (1988), 57, 358–363 © The Macmillan Press Ltd., 1988

### Expression of oncogenes in thyroid tumours: Coexpression of c-*erbB2/neu* and c-*erbB*

R. Aasland[1], J.R. Lillehaug[2], R. Male[2], O. Jøsendal[3], J.E. Varhaug[3] & K. Kleppe[1]

[1]*Laboratory of Biotechnology,* [2]*Department of Biochemistry and* [3]*Department of Surgery, University of Bergen, N-5029 Bergen, Norway.*

**Summary** The receptor-type oncogenes c-*erbB2/neu* and c-*erbB* have been found amplified and/or overexpressed in a number of tumours of epithelial origin. We have studied the expression of oncogenes in biopsies from human thyroid tumours. The c-*erbB2/neu* and c-*erbB* oncogenes showed two- to three-fold higher levels of RNA in papillary carcinomas and lymph node metastases as well as in one adenoma when compared to non-tumour tissue. The nuclear oncogenes c-*myc* and c-*fos* were found to be expressed at varying levels in both non-tumour and tumour tissue. RNA transcripts specific for the platelet-derived growth factor A and B chains and the N-*ras* oncogene were detected in one anaplastic carcinoma. Neither rearrangements nor amplifications of oncogenes were observed in the thyroid tumours. These data are particularly interesting in light of the recent findings that epidermal growth factor induces proliferation and dedifferentiation of normal thyroid epithelial cells *in vitro*. We suggest that the epidermal growth factor or other ligands for the c-*erbB* and c-*erbB2/neu* receptors may contribute to the development and/or maintenance of the malignant phentotype of papillary carcinomas of the thyroid.

Strong evidence has accumulated supporting the hypothesis that abnormalities in either the structure or activity of proto-oncogenes contribute to the development and/or maintenance of the malignant phenotype. The expression of oncogenes has been investigated in several types of human malignancies, most extensively by Slamon *et al.* (1984). In some cases tumour aggressivity and state of differentiation have been correlated to the expression of certain oncogenes. The N-*myc* oncogene has been found abundantly expressed in poorly differentiated regions of neuroblastomas (Schwab, 1985).

The c-*erbB* oncogene encodes the receptor for epidermal growth factor (EGF) (Downward *et al.*, 1984) and transforming growth factor alpha (TGF-$\alpha$) (Todaro *et al.*, 1980). The more recently discovered c-*erbB2/neu* oncogene is distinct from, but closely related to c-*erbB* and encodes a receptor-like glycoprotein with tyrosine kinase activity for which a ligand has not yet been identified (Bargmann *et al.*, 1986; Akiyama *et al.*, 1986; Yamamoto *et al.*, 1986*a*).

The c-*erbB* oncogene has been found overexpressed and/or amplified in a number of cancers of epithelial origin such as squamous carcinomas (Yamamoto *et al.*, 1986*a,b*) and in brain tumours (Libermann *et al.*, 1985). Elevated expression of the c-*erbB2/neu* gene has been reported to accompany the amplification in several instances (King *et al.*, 1985; Fukushige *et al.*, 1986; Yokota *et al.*, 1986; Kraus *et al.*, 1987). Recently, the c-*erbB2/neu* oncogene was found amplified in a large number of mammary carcinomas, and a strong correlation between c-*erbB2/neu*-amplification and poor prognosis of the disease was observed (Slamon *et al.*, 1987). The c-*fos* and c-*myc* oncogenes have been found expressed at elevated levels in a wide range of human tumours (Slamon *et al.*, 1984).

The study of oncogene expression in human tumours may thus become an important tool in the diagnosis and the evaluation of prognosis of specific types of malignant tumours. Thyroid tumours have been largely excluded from the surveys of oncogene expression in human tumours, probably due to the low incidence and low mortality from thyroid cancer. Only recently, evidence was presented for the existence of a new transforming gene in thyroid papillary carcinomas and their lymph node metastases (Fusco *et al.*, 1987). Additionally, thyroid tumours are of particular interest; firstly, due to the existence of a wide spectrum of growth abnormalities, both hyperplastic and neoplastic which are commonly subjected to surgical removal. Secondly, thyroid cancer displays non-random geographical distribution. In Norway, it is most frequent in the coastal districts (Glattre *et al.*, 1985). Furthermore it will be of importance to investigate the possible involvement of oncogenes in the trophic hormone control of the thyroid.

In this paper we report data on the expression of the receptor-type oncogenes c-*erbB* and c-*erbB2/neu*, and the nuclear oncogenes c-*fos* and c-*myc*, in thyroid tumours. Our data show that c-*myc* and c-*fos* are expressed abundantly in several of the tumours as well as in non-tumour tissue. In contrast, the highest levels of c-*erbB* and c-*erbB2/neu* RNA expression were found in papillary carcinomas and their lymph node metastases. In the tumours from the patients so far tested, neither gross rearrangements nor amplifications of oncogenes have been detected.

### Materials and methods

### *Enzymes and chemicals*

Restriction enzymes were from New England Biolabs. DNA polymerases and [$\alpha$-$^{32}$P]dATP and [$\alpha$-$^{32}$P]dCTP (3000 Ci mmol$^{-1}$) were from Amersham. Nitrocellulose membranes (BA-85) were from Schleicher and Schuell. Guanidinium thiocyanate was from Bethesda Research Laboratories. All other chemicals were of pro analytical grade.

### *Tissue samples*

Biopsies were collected from surgically removed thyroid lobes of patients subjected to either partial or total thyroidectomy. The samples consisted of both primary tumour tissue (5 papillary carcinomas, 1 anaplastic carcinoma, 2 adenomas) and 3 lymph node metastases as well as 5 samples of non-tumour tissue, including one from a diffuse toxic goitre. For comparison, a lymphoma of the thyroid, a mammary carcinoma, and a full term placenta from a healthy woman were included in the studies (see Table I for further data on the patients). DNA from a third adenoma (patient no. 2) was included in the Southern analysis only. Histological classification was performed by the Department of Pathology, The Gade Institute, University of Bergen. All the patients from whom thyroid tissues were studied, were euthyroid. Three of these patients (nos. 5, 7

Correspondence: R. Aasland.
Received 4 June 1987; and in revised form, 6 November 1987.

**Table I** Patient data

| *Patient no.*[a] | *Age* | *Sex* | *Diagnosis* |
|---|---|---|---|
| 2 | 31 | F | Adenoma |
| 5 | 17 | F | Papillary carcinoma |
| 7 | 23 | F | Diffuse toxic goitre |
| 8 | 37 | F | Adenoma |
| 12 | 81 | F | Papillary carcinoma |
| 13 | 35 | M | Papillary carcinoma |
| 14 | 37 | M | Adenoma (Hürtle cell) |
| 15 | 43 | F | Papillary carcinoma |
| 17 | 36 | F | Papillary carcinoma |
| 19 | 85 | M | Anaplastic carcinoma |
| Ly | 83 | F | Lymphoma of the thyroid |
| Mc | 77 | F | Mammary carcinoma |

[a]Tissue samples from each patient are denoted by the patient number followed by a sample number.

and 17) were on thyroxin (0.10–0.20 mg per day) for 5–32 weeks prior to surgery. Patient no. 7 was also given a thyrostatic drug (carbimazole; 30 mg per day, 28 weeks prior to surgery).

*Purification of DNA and RNA from biopsies*

Tissue samples taken from thyroid glands or lymph node metastases immediately after surgical removal from patients were frozen in liquid nitrogen without delay and thereafter stored at −80°C until further processing. DNA and total cellular RNA were simultaneously purified from the biopsies using a modification of the method described by Chirgwin *et al.* (1978) which was briefly as follows: The frozen tissue was minced briefly (max. 30 sec) at 0°C and immediately transferred to the guanidinium thiocyanate homogenization solution. The sample was homogenized in a Dounce's homogenizer, filtered through cheesecloth, added 1 g CsCl/2.5 ml and layered on top of a 1.25 ml cushion of 5.7 M CsCl in 0.1 M EDTA. Precipitation of RNA and banding of DNA was performed by centrifugation at 20°C for 12 h at 35,000 rpm in a Beckman SW50 rotor. DNA was extracted twice in phenol/chloroform and once in chloroform, ethanol-precipitated, and dissolved in 10 mM Tris-HCl/1 mM EDTA/pH 7.5. The RNA-pellet was dissolved in 10 mM Tris-HCl/1 mM EDTA/1% SDS/pH 7.5, extracted once with chloroform/n-butanol (4:1), ethanol-precipitated, and stored at −20°C until further processing. RNA concentration was determined either spectrophotometrically or by detection of fluorescence at 590 nm in the presence of ethidium bromide (2.5 $\mu g\,ml^{-1}$ in PBS; excitation at 360 nm).

*Hybridization analysis*

Restriction enzyme digested DNA was separated by electrophoresis on 0.7% agarose gels and blotted onto nitrocellulose as described by Maniatis *et al.* (1982). RNA was slot-blotted onto nitrocellulose as described by Murnane (1986) except that denaturation of RNA was carried out in 12% formaldehyde/105 mM sodiumphosphate/105 mM EDTA/pH 6.5. RNA was separated by electrophoresis in 1.1% agarose gels in a MOPS-buffer system in the presence of 2.2 M formaldehyde and blotted onto nitrocellulose. Hybridization to immobilized nucleic acids was performed as described by Maniatis *et al.* (1982), in the presence of 50% formamide/5 × SSC/200 $\mu g\,ml^{-1}$ heat-denatured salmon sperm DNA/0.1% SDS/25 mM sodiumphosphate pH 6.5/8.25% dextransulphate at 42°C for 15 to 24 h. Filters were washed twice for 20 min in 0.2 × SSC/0.1% SDS at 65°C. When filters were to be rehybridized, the bound probe was first removed by incubation of the filters in 0.1 × SSC for 7 min at 95–100°C. Kodak XAR-5 X-ray films were exposed to the nitrocellulose membranes at −80°C in the presence of intensifying screens. Densitometric scanning of the films was carried out using a Zeineh soft laser (Biomed Instruments Inc.) fitted with a HP3390A integrator (Hewlett Packard).

*Preparation of hybridization probes*

DNA fragments were prepared from plasmids and $^{32}P$-labelled by nick translation (Rigby *et al.*, 1977) or using the oligo-labelling technique (Feinberg & Vogelstein, 1984) resulting in specific activities ranging from 0.4 to $4 \times 10^9$ cpm $\mu g^{-1}$. The probes were purified fragments of cloned human genes. c-*fos*: 3 kbp *Xho*I-*Nco*I fragment of pc-*fos*-1 (Curran *et al.*, 1983); c-*myc*: 1.5 kbp *Cla*I-*Eco*RI fragment of pE-H8 (Gazin *et al.*, 1984); c-*erb*B2/*neu*: 0.44 kbp *Bam*HI fragment of pKXO44 (Semba *et al.*, 1985) and partial cDNA: 1.6 kbp *Eco*RI fragment of pCER204 (Yamamoto *et al.*, 1986*a*); c-*erbB* cDNA: 2.4 kbp *Cla*I fragment of pE7 (Xu *et al.*, 1984); N-*ras* partial cDNA: 0.55 kbp *Eco*RI-*Sal*I fragment of p6a1 (Taparowsky *et al.*, 1983); PDGF-A cDNA: 1.3 kbp *Eco*RI fragment of pUC-13-D1 (Betsholz *et al.*, 1986); v-*sis*: 1.2 kbp *Pst*I fragment of pv-*sis* (Robbins *et al.*, 1981); thyroglobulin partial cDNA: 0.68 and 0.98 kbp *Pst*I fragments of phTgl (Brocas *et al.*, 1982); Mouse 18S rRNA end-labelled with $^{32}P$ was a generous gift from Anne M. Øyan, Department of biochemistry, University of Bergen.

### Results

*Analysis of oncogene expression in thyroid tumours*

Slot-blot hybridization was the method of choice for detection of oncogene RNA due to its simplicity and high sensitivity. Three sets of slot-blotted RNA samples were prepared. The first set was analyzed by hybridization using probes for c-*myc*, c-*fos*, c-*erbB2/neu*, thyroblobulin, tubulin and 18S ribosomal RNA (Figure 1). The second set of slot-blotted RNA samples were hybridized to probes for c-*fos*, c-*erbB2/neu* (Figure 2), and c-*sis*, PDGF-A, (Figure 3) and L-*myc* (not shown), and the third set was hybridized to probes for c-*myc*, c-*erbB* (Figure 2), and N-*ras* (Figure 3). The results from densitometric scanning of the autoradiograms are presented in Figure 4.

*Expression of* c-erbB2/neu *and* c-erbB

All thyroid biopsies contained c-*erbB2/neu* and c-*erbB* RNA. Three out of 5 papillary carcinomas (13.4, 15.2 and 17.3) and the 3 lymph node metastases (5.7, 13.5 and 15.3) expressed these oncogenes at 2- to 3-fold higher levels than non-tumour tissue. The highest level of c-*erbB2/neu* RNA was observed in one of the two adenomas (8.4) while the anaplastic carcinoma (19.1) expressed low levels of both c-*erbB2/neu* and c-*erbB* RNA.

The c-*erbB2/neu* probe used in the first experiment (Figure 1) had a low specific activity ($7 \times 10^8$ cpm $\mu g^{-1}$) and the signals are barely visible; high levels of c-*erbB2/neu* specific RNA in this experiment were, however, observed in the two metastases (5.7 and 13.5). These results were confirmed in the second hybridization experiment (Figure 2).

Among the non-thyroid biopsies, the mammary carcinoma (Mc) expressed more than 18-fold higher levels of c-*erbB2/neu* RNA than the non-tumour tissues, whereas c-*erbB* RNA was not detected. This tumour exhibited a more than 20-fold amplification of the c-*erbB2/neu* gene (Figure 5). The placenta expressed high levels of c-*erbB* but not c-*erbB2/neu*.

*Expression of* c-myc *and* c-fos

All thyroid biopsies contained high levels of c-*myc* and c-*fos* RNA. The anaplastic carcinoma (19.1) expressed highest level of c-*myc* RNA while a lymph node metastasis (15.3)

**Figure 1** Expression of the c-*myc*, c-*fos*, c-*erbB2*/*neu* oncogenes, thyroglobulin, and tubulin in 3 thyroid tumours, a thyroid lymphoma, and a term placenta from a healthy woman. Samples of 20 μg total RNA was slot-blotted onto nitrocellulose membranes and hybridized to the probes indicated as described in **Materials and methods**. An end-labelled mouse 18S rRNA was used as a probe to evaluate the amount of RNA applied to the filter. PC: Papillary carcinoma; LM: Lymph node metastasis; Ly: Lymphoma; P: Placenta.

**Figure 2** Expression of c-*myc*, c-*fos*, c-*erbB*, and c-*erbB2*/*neu* in biopsies from thyroid tumours and non-tumour thyroid tissue, a thyroid lymphoma, a mammary carcinoma and a term placenta from a healthy woman. Samples of 12 μg total RNA was slot-blotted onto nitrocellulose membranes and hybridized to the probes indicated. NT: non-tumour tissue; AD: adenoma; AC: anaplastic carcinoma; PC: papillary carcinoma; M: lymph node metastasis; Ly: lymphoma; Mc: mammary carcinoma; P: placenta; ○: RNA not applied. Sample no. 19.2 was disregarded due to loss of RNA during sample preparation.

exhibited highest level of c-*fos* RNA. Notably, several samples of non-tumour tissue contained high levels of c-*myc* and c-*fos* RNA. It should also be noted that elevated expression of c-*fos* was observed in the three papillary carcinomas (13.4, 15.2 and 17.3) expressing the highest levels of c-*erbB2*/*neu* and c-*erbB* specific RNA.

**Figure 3** Expression of c-*sis*, N-*ras* and PDGF-A specific RNA in an anaplastic carcinoma (19.1). The same RNA slot-blots (12 μg RNA per slot) as used in **Figure 2** have been rehybridized to the probes as indicated.

The integrity of the RNA was sufficiently maintained as indicated by the presence of the 2.2 and 2.0 kb c-*fos* transcripts on a Northern blot (Figure 6). The low sensitivity in this experiment allowed detection of transcripts only in the four samples from Patients no. 12 and 13. Slight smearing of the *fos*-transcripts was apparent, indicating that some degradation of RNA had occured. This had most probably taken place during the lengthy and complicated surgical removal of the thyroid. A slight degradation of RNA should not, however, seriously affect the detection of slot blotted RNA.

*Expression of other genes*

N-*ras*, c-*sis* and PDGF-A specific RNA was expressed at low but invariant levels in all the thyroid samples (data not shown) except in the anaplastic carcinoma (19.1). This tumour showed 4-fold higher levels of N-*ras* and PDGF-A RNA as well as traces of c-*sis* RNA than the other thyroid samples as determined by densitometric scanning of autoradiograms (Figure 3). This tumour may therefore produce homo- as well as heterodimers of PDGF. L-*myc* expression was not detected in any of the samples tested (not shown).

Thyroglobulin specific RNA was expressed at very high levels in all the thyroid samples while being completely absent from the lymphoma and the placenta (Figure 1). To serve as an internal control, the filter was rehybridized to a tubulin and a mouse 18S rRNA probe (Figure 1). The results clearly demonstrate that the level of tubulin RNA varied to a great extent among the tissue specimens and thus was of little value as an internal control. The 18S rRNA hybridization indicated that the amount of RNA applied to the filter varied only slightly.

*Southern blot-hybridization to tumour DNA*

In Figure 6 the Southern blot analysis of various thyroid tumour DNAs using the c-*erbB2*/*neu* probe is shown. Neither amplification nor rearrangements were found. In contrast, the c-*erbB2*/*neu* gene was amplified more than 20-fold in the mammary carcinoma (Mc).

Restriction fragment analyses of the c-*myc*, c-*fos*, and c-*erbB* oncogenes as well as others (including c-*myb*, c-*etc-1*, c-*src*, *int-1* and p53) have also been carried out with DNA from the thyroid tumours. No rearrangements nor amplifications have so far been detected (results not shown).

### Discussion

We have studied the expression of oncogenes in fresh biopsies from 8 patients having different types of thyroid tumours and in biopsies from a diffuse toxic goitre and a lyphoma of the thyroid. The expression of the c-*erbB* and c-*erbB2*/*neu* oncogenes was found to be 2- to 3-fold higher in 3 of 5 papillary carcinomas, 3 lymph node metastases, and one adenoma than in non-tumour tissue and the anaplastic

**Figure 4** Relative levels of oncogene expression as measured by densitometric scanning of the autoradiograms shown in **Figures 1 and 2**. The units of expression are arbitrary. The biopsies are grouped according to histological classification as follows: NT: non-tumour; PC: Papillary carcinoma; AC: Anaplastic carcinoma; AD: Adenoma; OT: other tissues; P: Placenta; Ly: Lymphoma; Mc: Mammary carcinoma. (nd: not determined).

carcinoma. Our data extend the list of human tumours in which an elevated expression of the c-*erbB2/neu* and c-*erbB* oncogenes is found also to include papillary carcinomas of the thyroid. To decide whether this is a feature of these carcinomas in general, will require further data. The expression of c-*erbB2/neu* in the thyroid samples was moderate as compared to the mammary carcinoma that contained a >20-fold amplified c-*erbB2/neu* gene. Intermediate level expression of these receptor type oncogenes may, nevertheless, be important. The A431 epidermoid carcinoma cells which are expressing very high levels of EGF-receptors have been found to be inhibited by EGF at concentrations which are mitogenic to other cells (Gill & Lazar, 1981; Barnes, 1982). An elevated expression of these oncogenes at the RNA level does not imply that the cells express higher levels of functional receptors, although this is most likely. The expression of c-*erbB* was clearly evident in non-tumour thyroid tissue. This finding is supported by the observation by Humphries *et al.* (1983) that normal thyroid epithelial cells possess EGF-receptors. It has recently been shown by several groups that EGF induces proliferation and dedifferentiation of normal thyroid epithelial cells in culture (Eggo *et al.*, 1984; Roger & Dumont, 1984; Westermark *et al.*, 1983; Waters *et al.*, 1987). Thus it is possible that an elevated expression of EGF-receptors contributes to the development of a malignant phenotype of thyroid tumours by increasing proliferation and dedifferentiation. Pertinent to this are the reports on the induction by EGF of plasminogen activator which in turn may lead to the degradation of extracellular matrix proteins (Lee & Weinstein, 1978; Stoppelli *et al.*, 1986). In mammary carcinomas, there is now evidence for the expression of TGF-α and its possible involvement in an autocrine or paracrine stimulation of growth (Dickson *et al.*, 1987). An increased level of EGF-receptors may potentiate such stimulation. We are currently investigating whether TGF-α is expressed in the thyroid tumours. Although EGF did not stimulate the tyrosine kinase activity of the c-*erbB2/neu* protein, EGF induced tyrosine and serine phosphorylation of the c-*erbB2/neu* protein (Akiyama *et al.*, 1986; Kadowaki *et al.*, 1987). This suggests that the coexpression of the c-*erbB2/neu* and c-*erbB* oncogenes in the thyroid tumours demonstrated in the present report, may have functional relevance.

The c-*myc* and c-*fos* oncogenes were expressed at varying levels in tumour tissue as well as in non-tumour tissue. There was no correlation with the expression of these two oncogenes and the type of thyroid tumour. High levels of c-*myc* and c-*fos* RNA have been reported in a wide range of human tumours (Slamon *et al.*, 1984). Our data add thyroid tumours to this list. The presence of elevated levels of c-*myc* and c-*fos* RNA in the non-tumour tissue samples indicates that expression of these genes does not imply malignancy. One should note, however, that the non-tumour biopsies were taken from tumour-bearing thyroids (except the one from a diffuse toxic goitre). It is possible that the entire tumour-bearing thyroids were in a state of growth-stimulation that could induce the expression of c-*myc* and c-*fos* as has been shown *in vitro* (Dere *et al.*, 1985; Tramontano *et al.*, 1986; Colletta *et al.*, 1986). Furthermore, the expression of these genes may be unevenly distributed in the tumours, and we are currently investigating this possibility by means of *in situ* hybridization.

The anaplastic carcinoma exhibited a markedly different pattern of oncogene expression when compared to the papillary carcinomas. In particular, the c-*myc* and N-*ras* oncogenes were expressed at high levels. The anaplastic carcinoma may be an example of a tumour in which the action of these two oncogenes contributes to the transformation in a cooperative manner (Land *et al.*, 1983). This tumour also expressed RNA specific for the A and B chains of PDGF. These differences may reflect the different properties of these two types of tumours, the anaplastic carcinomas being far more aggressive than the papillary carcinomas.

In a separate line of studies using oligonucleotide

**Figure 5** Southern blot of DNA from 4 thyroid tissue specimens, a mammary carcinoma, and a placenta hybridized to a c-*erbB2/neu* probe. Size markers are phage lambda *Hin*dIII fragments. P: Placenta; Mc: Mammary carcinoma.

**Figure 6** Hybridization with the c-*fos* probe to a Northern blot ot total cellular RNA from four samples of thyroid tumour tissue. The sizes of the transcripts were determined relative to radioactively labelled phage lambda *Hin*dIII fragments.

hybridization, we have looked for point-mutations in codon 12 of the c-K-*ras* oncogene in the thyroid tumours. No such mutations have been detected (Rusken & Aasland, unpublished observations). Neither have we found any gross rearrangements not amplifications of oncogenes in the thyroid tumours investigated and, in particular, no amplifications of the c-*erbB* and c-*erbB2/neu* oncogenes were observed. The deregulation of these genes in thyroid tumours may thus be due to molecular mechanisms other than genetic alterations of these genes. Kraus and coworkers (1987) also observed elevated expression of the c-*erbB2/neu* gene in several mammary tumour cell lines that did not exhibit an amplified gene.

Further studies on the expression and regulation of the c-*erbB* and c-*erbB2/neu* genes in malignant and non-malignant thyroid tumours will shed light on the role of these oncogenes in the development and maintenance of a malignant phenotype.

We thank Drs Dominique Stehlin, Tom Curran, Gilbert Vassart, Tadashi Yamamoto, Christer Betsholtz, Michael Wigler and Ira Pastan for sharing probes with us, and Jim B. Lorens for excellent technical assistance. This work was supported by the Norwegian Cancer Society and the Faculty of Medicine, University of Bergen. R.A. and R.M. are fellows of the Norwegian Cancer Society.

### References

AKIYAMA, T., SUDO, C., OGAWARA, H., TOYOSHIMA, K. & YAMAMOTO, T. (1986). The product of the human c-*erb*B-2 gene: A 185-kilodalton glycoprotein with tyrosine kinase activity. *Science*, **232**, 1644.

BARGMANN, C.I., HUNG, M.-C. & WEINBERG, R.A. (1986). The *neu* oncogene encodes an epidermal growth factor receptor-related protein. *Nature*, **319**, 226.

BARNES, D.W. (1982). Epidermal growth factor inhibits growth of A431 epidermoid carcinoma in serum-free cell culture. *J. Cell Biol.*, **93**, 1.

BETSHOLTZ, C., JOHNSSON, A., HELDIN, C.-H. & 9 others (1986). cDNA sequence and chromosome localization of human platelet-derived growth factor A-chain and its expression in tumor cell lines. *Nature*, **320**, 695.

BROCAS, H., CHRISTOPHE, D., POHL, V. & VASSART, G. (1982). Cloning of human thyroglobulin complementary DNA. *FEBS Lett.*, **137**, 189.

CHIRGWIN, J.M., PRZYBYLA, A.E., MACDONALD, R.J. & RUTTER, W.J. (1979). Isolation of biologically active ribonucleic acid from sources enriched in ribonuclease. *Biochemistry*, **18**, 5294.

COLLETTA, G., CIRAFICI, A.M. & VECCHIO, G. (1986). Induction of the c-*fos* oncogene by thyrotropic hormone in rat thyroid cells in culture. *Science*, **233**, 458.

CURRAN, T., MACCONNELL, W.P., VAN STRATEN, F. & VERMA, I.M. (1983). Structure of the FBJ Murine Osteosarcoma virus genome: Molecular cloning of its associated helper virus and the cellular homolog of the v-*fos* gene from mouse and human cells. *Mol. Cell. Biol.*, **3**, 914.

DICKSON, R.B., KASID, A., HUFF, K.K. & 5 others (1987). Activation of growth factor secretion in tumorigenic states of breast cancer induced by 17β-estradiol or v-Ha-*ras* oncogene. *Proc. Natl Acad. Sci. USA*, **84**, 837.

DERE, W.H., HIRAYU, H. & RAPOPORT, B. (1985). TSH and cAMP enhance expression of the *myc* proto-oncogene in cultured thyroid cells. *Endocrinol.*, **117**, 2249.

DOWNWARD, J., YARDEN, Y., MAYES, E. & 6 others (1984). Close similarity of the epidermal growth factor receptor and v-*erb*-B oncogene protein sequences. *Nature*, **307**, 521.

EGGO, M.C., BACHRACH, L.K., FAYET, G. & 4 others (1984). The effects of growth factors and serum on DNA synthesis and differentiation in thyroid cells in culture. *Mol. Cell. Endocrinol.*, **38**, 141.

FEINBERG, A.P. & VOGELSTEIN, B. (1984). A technique for radiolabeling DNA restriction endonuclease fragments to high specific activity. *Anal. Biochem.*, **137**, 266.

FUKUSHIGE, S.-I., MATSUBARA, K.-I., YOSHIDA, M. & 5 others (1986). Localization of a novel v-*erb*B-related gene, c-*erb*B-2, on human chromosome 17 and its amplification in a gastric cancer cell line. *Mol. Cell. Biol.*, **6**, 955.

FUSCO, A., GRIECO, M., SANTORO, M. & 5 others (1987). A new oncogene in human thyroid papillary carcinomas and their lymph-nodal metastases. *Nature*, **328**, 170.

GAZIN, C., DE DINECHIN, S., HAMPE, A. & 4 others (1984). Nucleotide sequence of the human c-*myc* locus: Provocative open reading frame within the first exon. *EMBO J.*, **3**, 383.

GILL, G.N. & LAZAR, C.S. (1981). Increased phosphotyrosine content and inhibition of proliferation in EGF-treated A431 cells. *Nature*, **293**, 305.

GLATTRE, E., FINNE, T.E., OLESEN, O. & LANGMARK, F. (1985). *Atlas of Cancer Incidence in Norway 1970–79*. The Norwegian Cancer Society and Cancer Registry of Norway: Oslo.

HUMPHRIES, H., MACNEIL, S., MUNRO, D.S. & TOMLINSON, S. (1983). Interaction of epidermal growth factor with receptors on human and porcine thyroid membranes. *J. Endocrinol.*, **102**, 57.

KADOWAKI, T., KASUGA, M., TOBE, K. & 8 others (1987). A Mr = 190,000 glycoprotein phosphorylated on tyrosine residues in epidermal growth factor stimulated KB cells is the product of the c-*erb*B-2 gene. *Biochem. Biophys. Res. Com.*, **144**, 699.

KING, C.R., KRAUS, M.H. & AARONSON, S.A. (1985). Amplification of a novel v-*erb*B-related gene in a human mammary carcinoma. *Science*, **229**, 974.

KRAUS, M.H., POPESCU, N.C., AMSBAUGH, C. & KING, C.R. (1987). Overexpression of the EGF receptor-related proto-oncogene *erb*B-2 in human mammary tumor cell lines by different molecular mechanisms. *EMBO J.*, **6**, 605.

LAND, H., PARADA, L.F. & WEINBERG, R.A. (1983). Tumorigenic conversion of primary embryo fibroblasts requires at least two cooperating oncogenes. *Nature*, **304**, 596.

LEE, L.-S. & WEINSTEIN, I.B. (1978). Epidermal growth factor, like phorbol esters, induces plasminogen activator in HeLa cells. *Nature*, **274**, 696.

LIBERMANN, T.A., NUSBAUM, H.R., RAZON, N. & 7 others (1985). Amplification, enhanced expression and possible rearrangement of EGF receptor gene in primary human brain tumours of glial origin. *Nature*, **313**, 144.

MANIATIS, T., FRITSCH, E.F. & SAMBROOK, J. (1982). *Molecular Cloning*. Cold Spring Harbor Laboratory: New York.

MURNANE, J.P. (1986). Inducible gene expression by DNA rearrangements in human cells. *Mol. Cell. Biol.*, **6**, 549.

RIGBY, P.W.J., DIECKMANN, M., RHODES, C. & BERG, P. (1977). Labeling deoxyribonucleic acid to high specific activity *in vitro* by nick translation with DNA polymerase I. *J. Mol. Biol.*, **113**, 237.

ROBBINS, K.C., DEVARE, S.G. & AARONSON, S.A. (1981). Molecular cloning of integrated simian sarcoma virus: Genome organization of infectious DNA clones. *Proc. Natl Acad. Sci. USA*, **78**, 2918.

ROGER, P.P. & DUMONT, J.E. (1984). Factors controlling proliferation and differentiation of canine thyroid cells cultured in reduced serum conditions: Effects of thyrotropin, cyclic AMP and growth factors. *Mol. Cell. Endocrinol.*, **36**, 79.

SCHWAB, M. (1985). Amplification of N-*myc* in human neuroblastomas. *Trend. Genet.*, **1**, 271.

SEMBA, K., KAMATA, K., TOYOSHIMA, K. & YAMAMOTO, T. (1985). A v-*erb*B related protooncogene, c-*erb*B-2, is distinct from the c-*erb*B-1/EGF receptor gene and is amplified in a human salivary gland adenocarcinoma. *Proc. Natl Acad. Sci. USA*, **82**, 6497.

SLAMON, D.J., DE KERNION, J.B., VERMA, I.M. & CLINE, M.J. (1984). Expression of cellular oncogenes in human malignancies. *Science*, **224**, 256.

SLAMON, D.J., CLARK, G.M., WONG, S.G., LEVIN, W.J., ULLRICH, A. & MCGUIRE. (1987). Human breast cancer: Correlation of relapse and survival with amplification of the HER-2/*neu* oncogene. *Science*, **235**, 177.

STOPPELLI, M.P., VERDE, P., GRIMALDI, G., LOCATELLI, E.K. & BLASI, F. (1986). Increase in urokinase plasminogen activator mRNA synthesis in human carcinoma cells is a primary effect of the potent tumor promoter, phorbol myristate acetate. *J. Cell. Biol.*, **102**, 1235.

TAPAROWSKY, E., SHIMIZU, K., GOLDFARB, M. & WIGLER, M. (1983). Structure and activation of the human N-*ras* gene. *Cell*, **34**, 581.

TODARO, G.J., FRYLING, C. & DE LARCO, J.E. (1980). Transforming growth factors produced by certain human tumor cells: Polypeptides that interact with epidermal growth factor receptors. *Proc. Natl Acad. Sci. USA*, **77**, 5258.

TRAMONTANO, D., CHIN, W.W., MOSES, A.C. & INGBAR, S.H. (1986). Thyrotropin and dibuturyl cyclic AMP increase levels of c-*myc* and c-*fos* mRNA in cultured rat thyroid cells. *Jour. Biol. Chem.*, **261**, 3919.

WATERS, M.J., TWEEDALE, R.C., WHIP, T.A., SHAW, G., MANLEY, S.W. & BOURKE, J.R. (1987). Dedifferentiation of cultured thyroid cells by epidermal growth factor: Some insights into the mechanism. *Mol. Cell. Endocrinol.*, **49**, 109.

WESTERMARK, K., KARLSSON, F.A. & WESTERMARK, B. (1983). Epidermal growth factor modulates thyroid growth and function in culture. *Endocrinol.*, **112**, 1680.

XU, Y.-H., ISHII, S., CLARK, A.J.L. & 6 others (1984). Human epidermal growth factor receptor cDNA is homologous to a variety of RNAs overproduced in A431 carcinoma cells. *Nature*, **309**, 806.

YAMAMOTO, T., IKAWA, S., AKIYAMA, T. & 5 others (1986*a*). Similarity of protein encoded by the human c-*erb*B-2 gene to epidermal growth factor receptor. *Nature*, **319**, 230.

YAMAMOTO, T., KAMATA, N., KAWANO, H. & 9 others (1986*b*). High incidence of amplification of the epidermal growth factor receptor gene in human squamous carcinoma cell lines. *Cancer Res.*, **46**, 414.

YOKOTA, J., YAMAMOTO, T., TOYOSHIMA, K. & 4 others (1986). Amplification of c-*erb*B-2 oncogene in human adenocarcinomas *in vivo*. *Lancet*, **i**, 765.